# QLCA and Entangled States as Single-Neuron Activity Generators

**DOI:** 10.3389/fncom.2021.600075

**Published:** 2021-06-02

**Authors:** Yehuda Roth

**Affiliations:** Oranim Academic College, Science Department, Kiryat Tiv'on, Israel

**Keywords:** eigenconcepts, quantum cellular automata, quantum observer, QCA operators, entanglement, collapse, single neuron activity

## Abstract

Each neuron in the central nervous system has many dendrites, which provide input information through impulses. Assuming that a neuron's decision to continue or stop firing is made by rules applied to the dendrites' inputs, we associate neuron activity with a quantum like-cellular automaton (QLCA) concepts. Following a previous study that related the CA description with entangled states, we provide a quantum-like description of neuron activity. After reviewing and presenting the entanglement concept expressed by QLCA terminology, we propose a model that relates quantum-like measurement to consciousness. Then, we present a toy model that reviews the QLCA theory, which is adapted to our terminology. The study also focuses on implementing QLCA formalism to describe a single neuron activity.

## 1. Introduction

It is recognized that brain activities such as thoughts, actions, and percepts are a result of the simultaneously coordinated activities of many neurons Cooper (1973); Hopeld (1982). Since the microscopic approach that analyzes the internal interactions system is complex for analysis, scaling methods such as the coarse-graining procedures are implemented (Meshulam et al., 2019). We assume that once a scaling approach succeeds in describing the system by “macroscopic” rules, cellular automaton (C.A) techniques can be implemented.

CA is a set of rules that need to be followed by the components of a system to define a discrete time evolution by using generations of error corrections. A quantum CA process (QCA) integrates quantum concepts with CA; that is, it refers to quantum systems that evolve according to the CA rules (Arrighi, 2019; Farrelly, 2019). Studies have shown that it is possible to define entangled states that represent all alternatives before implementing the CA rule (Roth, 2019a; Roth and Roth, 2021), and considering entanglement as an exclusive quantum phenomenon, CA systems described by entangled states are considered as quantum systems.

Recently, a classical analogy of entanglement was suggested using classical light beams (Spreeuw, 1998; Chitambar and Gour, 2019; Friis et al., 2019; Konrad and Forbes, 2019). Hence, the traditional approach of viewing entangled states as a pure quantum phenomenon was refuted. Consequently, based on Roth (2019a) we can now say that whenever a system evolution is determined by a CA process, the corresponding rules associated with the entangled state need not be related to quantum mechanics. This enables the implementation of a quantum-like technique on a non-quantum system, such as the brain, thereby referring to the associated mathematical framework as QLCA.

In this paper, we describe neuron behavior through a QLCA process. Since a neuron decision of firing or halting follows some rules imposed by various incoming dendritic pulses, we associate each neuron decision with QLCA rules (Wolfram, 1983; Schiff, 2011; Beiki and Shahidinejad, 2014), which can be associated to a concept according to our description. In other words, by implementing the entangled states description, we assume that the multi-neuron pulses that arrive to an observed neuron are already arranged as a kind of concept. Models associating CA for studying brain activities have already been proposed, but in different contexts. In Fraile et al. (2018), the CA Game of Life model was implemented. Other CA models have been used in several problems related to neuroscience (Hopfield, 1982; Hoffman, 1987; Ermentrout and Edelstein-Keshet, 1993; Kansal et al., 2000; Tsoutsouras et al., 2012; Acedo et al., 2015). An extensive approach was presented in Gilpin (2019), where the CA rules were implemented between different layers of convolutional neural networks (Aloysius and Geetha, 2017).

The present paper presents the corresponding mathematical formalism, thereby introducing operators that describe brain activities. In our model, the encryption of information that ascends from the subconsciousness to the consciousness is related to an observable operator that detects and interprets this information (relations between quantum measurement and interpretation are shown in Roth, 2013a,b, 2017). Furthermore, some subconsciousness activities are described by unitary operators that correct processes that may disobey the CA rules.

## 2. Entanglement as Concept Generation

In this section, we demonstrate the relation between entangled states to concepts, where the term “concept” is associated with an entangled state. This term has not been used in previous studies, but it is suitable for our formalism. However, scaling models such as coarse-graining, it can refer to the dimensionality reduction of the system into a cluster (Meshulam et al., 2019). At this stage, we present the entangled states as another description for macroscopic clusters in the coarse graining model. In this approach, statistical models can be replaced by the operator formulation.

In this section, we use the quantum terminology of particles.

Suppose that we have *N* particles with two states |0〉 or |1〉, which will later be associated with the firing or misfiring of a neuron. A possible basis for states to span the Hilbert space is the distinguishable states:

(1) $$\begin{array}{l} |\psi \rangle =\overset{N}{\underset{i=1}{\prod }}|k_{\psi }\rangle_{i},\;\psi =1,2,\ldots ,2^{N} \end{array}$$

where *k* can have the values 0 or 1, and ψ denotes a set of {*k*_ψ_} combinations out of the 2^*N*^ possible products. To bridge the gap between the “descriptive brain terminologies” and the operator-oriented mathematical language, we use the descriptive term “concept.” For example, if we have an entangled state,

(2) $$\begin{array}{l} |=\rangle =\frac{1}{\sqrt{2}}\{\overset{N}{\underset{i=1}{\prod }}|0\rangle_{i}+\overset{N}{\underset{i=1}{\prod }}|1\rangle_{i}\} \end{array}$$

regardless of the specific individual state |0〉 or |1〉, the appropriate concept will be *the same*.

Another example is when we divide the states into subgroups with various concepts, such as the following pairs:

**The** *i* **and the** *i* + 1 **items are always in opposite states**

(3) $$\begin{array}{l} |\neq \rangle =\frac{1}{\sqrt{2}}\{|0\rangle_{i}|1\rangle_{i+1}+|1\rangle_{i}|0\rangle_{i+1}\}, \end{array}$$

**The** *i* **and the** *i* + 1 **items are always in the same states**

(4) $$\begin{array}{l} |=\rangle =\frac{1}{\sqrt{2}}\{|0\rangle_{i}|0\rangle_{i+1}+|1\rangle_{i}|1\rangle_{i+1}\}, \end{array}$$

Thus, it is seen that, for many states, it is possible to generate many concepts that represent various relations between states.

In the next section, we introduce the mathematical formalism of measuring concepts. The term concept is represented by a mathematical feature *eigenconcept*. Using this terminology, we associate a concept measurement with a new approach, considering the ascendance of information from unconsciousness to consciousness as a quantum measurement process.

### 2.1. Consciousness Information Presented as an Observer Who Detects Eigenconcepts

Following the coarse-graining or dimensionality reduction approach, an approach was proposed for describing unconscious and conscious states related to the multiple microscopic configurations of cells arranged in clusters. For example, Velazquez et al. (2019) presents a possible framework that characterizes the dynamics of the nervous system and its organization in the conscious and unconscious states, derived from the perspective of brain cell ensembles.

The entanglement approach can be considered as a mathematical alternative for describing scaling methods where the entangled state replaces the coarse-graining clusters. However, we further implement quantum ideas to provide a new perspective for understanding the concepts of unconscious and conscious.

Embedding the quantum collapse into our formalism, we integrate our approach with a publication titled “Does Consciousness Cause Quantum Collapse?” which referred to “Radical Theories of Consciousness” (McQueen, 2017). This yields a mathematical framework for describing the concepts of consciousness and unconsciousness using an observer (consciousness) and a measured environment (unconsciousness).

The theory of quantum measurement introduces the collapse concept. That is, after macroscopic measurement, a microscopic system that was in a state different from the set of possible measurement outputs, collapses into one of the measuring device eigenstates (Ghirardi et al., 1986; Bassi et al., 2013; Pokorny et al., 2020; Bassi, 2021). Recently, it was suggested that non-quantum systems can also possess collapse scenarios. For example, the implementation of a CA rule corresponds to a collapse (Roth, 2019a). In addition, suggestions for a collapse in classical mechanics were introduced (Roth, 2019b). Considering these non-quantum collapses allows us to consider the collapse scenarios in brain activities.

Roughly speaking, subconsciousness activities can end by floating the subconsciousness content to recognized and interpreted in the consciousness part. In the same manner an observer recognizes reality through measurements, we suggest that content recognition in consciousness should be represented by an abstract observer who reveals the hidden unconsciousness content as outputs of quantum-like measurements.

The conventional mathematical platform for describing a measuring device is represented by a series of projecting operators, each multiplied by its individual eigenvalue. We extend this description by replacing the numerical eigenvalues with descriptive visual objects defined as *eigenconcepts*. These will serve as the descriptive output of the measurement.

To demonstrate this formalism, we consider *N* = 2 for the state of Equation (1) and consider the following entangled states:

**The states are always in the opposite states**

(5) $$\begin{array}{l} |\neq \rangle =\frac{1}{\sqrt{2}}\{|0\rangle_{1}|1\rangle_{2}+|1\rangle_{1}|0\rangle_{2}\}, \end{array}$$

**The states are always in the same states**

(6) $$\begin{array}{l} |=\rangle =\frac{1}{\sqrt{2}}\{|0\rangle_{1}|0\rangle_{2}+|1\rangle_{1}|1\rangle_{2}\}, \end{array}$$

Suppose that brain activity is focused on comparing two objects, that is, it implements the concepts “*the same*” or the “*opposite*.” Hence, the consciousness encryption corresponds to the observable.

(7) $$\begin{array}{l} 𝕆=“\neq “|\neq \rangle \langle \neq |+“=“|=\rangle \langle =| \end{array}$$

where the strings “ ≠ ” and “=” describe the role of eigenconcepts.

After adapting all aspects of the measurement theory and, in particular, the radical collapse phenomenon (Ghirardi et al., 1986; Bassi et al., 2013; Pokorny et al., 2020; Bassi, 2021), we can include the consciousness interpretation in our description (Roth, 2013a,b, 2017). If the subconsciousness generates a state that is a superposition of the possible measured states (i.e., a superposition of the eigenstates of the observable), Then, the uploaded consciousness information when measured will collapse into one of the eigenstates to form an interpretation (Roth, 2013a,b, 2017).

## 3. Toy Model

In the previous sections, we demonstrated how an entangled state represents a concept. Herein, we show how CA iterations generate entangled states. For simplicity, this process is demonstrated using a toy model.

### 3.1. Entangled States as a Representative of Quantum-like Cellular Automaton Rules

To understand the mechanism of a QLCA system, let us consider a simple model (toy model), as shown in Roth (2019a). Consider a one-dimensional grid of cells labeled by the index *i*. Each cell can be in the state |0〉 or |1〉. In our model, the evolution of the system was determined by the following rules:

- **Set of rules A**
  – Rule *a*_*I*_: An *i* cell will be in the state |1〉 if the neighboring cells are in different states
  – Rule *a*_*II*_: An *i* cell will be at |0〉 when both neighbors are in the same state.
- **Set of rules B**
  – Rule *b*_*I*_: An *i* cell will be in the state |0〉 if the neighboring cells are in different states
  – Rule *b*_*II*_: An *i* cell will be at |1〉 when both neighbors are in the same state.

An appropriate state to describe possible relations between cells is as follows:

- **Neighbors of the** ***i*****-cell are in different states**:
  (8) $$\begin{array}{l} |\neq \rangle_{i}\overset{\text{def}}{=}\frac{1}{\sqrt{2}}(|0\rangle_{i-1}|1\rangle_{i+1}+|1\rangle_{i-1}|0\rangle_{i+1}). \end{array}$$
  This implies that regardless of a specific cell's state, the neighboring states are always in different states.
- **Neighbors of the** ***i*****-cell are in the same states**:
  (9) $$\begin{array}{l} |=\rangle_{i}\overset{\text{def}}{=}\frac{1}{\sqrt{2}}(|0\rangle_{i-1}|0\rangle_{i+1}+|1\rangle_{i-1}|1\rangle_{i+1}). \end{array}$$
  This implies that regardless of a specific cell's state, the neighboring states are always in the same state.

This yields the **CA-state definition for an** ***i*****-cell**,

- **States for set A**
  (10) $$\begin{array}{l} Rule\;a_{I}\;|a_{I}\rangle_{i}=|1\rangle_{i}\cdot |\neq \rangle_{i},\\ Rule\;a_{II}\;|a_{II}\rangle_{i}=|0\rangle_{i}\cdot |=\rangle_{i}, \end{array}$$
- **States for set B**
  (11) $$\begin{array}{l} Rule\;b_{I}\;|b_{I}\rangle_{i}=|0\rangle_{i}\cdot |\neq \rangle_{i},\\ Ruleb_{II}\;|b_{II}\rangle_{i}=|1\rangle_{i}\cdot |=\rangle_{i}, \end{array}$$

### 3.2. QLCA Operators in the Toy Model

We distinguish between two types of operators:

- Correction operators: By observing a CA net at some time, we may find areas that disobey the CA rules. The purpose of the correction operations is to fix these errors. Note that fixing the local sites can cause new errors in other locations. These were fixed in the subsequent iterations. Thus, the system evolution is composed of the generation of correction phases. This regular procedure in CA systems is now expressed in terms of the correction operators. We consider the correcting operations as a subconsciousness process.
- Observable operators: These represent the state to an observer after the entire QLCA process is complete. Recall that we associate the observable part with consciousness activities, in which a concept is generated by the entangled output state.

For the toy model, the operators are as follows:

- **Correction operators**
  - **Set A**
    – **Local correction operator**
      (12) $$\begin{array}{l} {\mathbb{C}}_{i}^{\left\{A\right\}}=|a_{I}\rangle_{i}\langle b_{I}{|}_{i}+|a_{II}\rangle_{i}\langle b_{II}{|}_{i} \end{array}$$
    **– Global correction operator for** ***k*** **generations**
      (13) $$\begin{array}{l} {\mathbb{C}}_{n}^{\left\{A\right\}}={\left(\underset{i}{\sum }{\mathbb{C}}_{i}^{\left\{A\right\}}\right)}^{k} \end{array}$$
  - **Set B**
    – **Local correction operator for set B**
      (14) $$\begin{array}{l} {\mathbb{C}}_{i}^{\left\{B\right\}}=|b_{I}\rangle_{i}\langle a_{I}{|}_{i}+|b_{II}\rangle_{i}\langle a_{II}{|}_{i} \end{array}$$
    – **Global correction operator for** ***k*** **generations**
      (15) $$\begin{array}{l} {\mathbb{C}}_{n}^{\left\{B\right\}}={\left(\underset{i}{\sum }{\mathbb{C}}_{i}^{\left\{B\right\}}\right)}^{k} \end{array}$$
- **Observable:** Quantum measurement corresponds to a macroscopic system (the measuring device) that, when implemented on a quantum system (usually microscopic), collapses the detected state into one of the eigenstates of the measuring device (Ghirardi et al., 1986; Pokorny et al., 2020).
  According to many estimates, the human brain contains approximately 10^11^ neurons (Lent et al., 2012). Although not all neurons participate in a specific brain activity, many relevant neurons establish a so-called macroscopic ensemble that allows a quantum-like measurement. This includes collapses into eigenstates, or eigenconcepts, which can be referred to as brain interpretation, as described in Roth (2013a), Roth (2013b), and Roth (2017).
  Because all the rule states of Equations 10 and 11 are orthogonal, we can express an observable with the projecting operators:
  - Set A
    – **Local observable**
      (16) $$\begin{array}{l} {𝕆}_{i}^{\left\{A\right\}}=|a_{I}\rangle_{i}\langle a_{I}{|}_{i}+|a_{II}\rangle_{i}\langle a_{II}{|}_{i}. \end{array}$$
    – **Global observable for set A**
      (17) $$\begin{array}{l} {𝕆}^{\left\{A\right\}}=“\sigma_{a}“\underset{i}{\sum }{𝕆}_{i}^{\left\{A\right\}} \end{array}$$
  - Set B
    – **Local observable for set B**
      (18) $$\begin{array}{l} {𝕆}_{i}^{\left\{B\right\}}=|b_{I}\rangle_{i}\langle b_{I}{|}_{i}+|b_{II}\rangle_{i}\langle b_{II}{|}_{i} \end{array}$$
    – **Global observable for set B**
      (19) $$\begin{array}{l} {𝕆}^{\left\{B\right\}}=“\sigma_{b}“\underset{i}{\sum }{𝕆}_{i}^{\left\{B\right\}} \end{array}$$

Note that neither the states |*a*_*I*_〉_*i*_ and |*a*_*II*_〉_*i*_ nor the states |*b*_*I*_〉_*i*_ and |*b*_*II*_〉_*i*_ form a complete set. Therefore, we have $\underset{i}{\sum }{𝕆}_{i}^{\left\{A\right\}}\neq 1$ and $\underset{i}{\sum }{𝕆}_{i}^{\left\{B\right\}}\neq 1$ to obtain a non-trivial expression for the observable.

To describe the measurement outputs, we use *eigenconcepts* “σ_*a*_” and “σ_*b*_” instead of numeric eigenvalues. These symbols can serve as a mathematical platform to describe how an observer experiences a concept.

Without concrete data on expressing total brain activity by operator terms, many models can be proposed. We suggest the following model. First, a type of set is selected, say, set A. The system runs ℂ *k* times in what we associate with an unconscious activity; then, by implementing 𝕆, a measurement is conducted to float a consciousness result. The associated operators are as follows:

(20) $$\begin{array}{l} {𝔸}_{k}={𝕆}^{\left\{A\right\}}{\left({\mathbb{C}}^{\left\{A\right\}}\right)}^{k}\\ {𝔹}_{k}={𝕆}^{\left\{B\right\}}{\left({\mathbb{C}}^{\left\{B\right\}}\right)}^{k} \end{array}$$

As mentioned, other models are legitimate. We leave this to future research.

## 4. QLCA and Brain Activity

In previous sections, we introduced simple scenarios of two-state system and a toy model to demonstrate a phenomenological procedure in the brain, described within the QLCA model. We introduced two operators: correcting operators that fix possible errors, and observable operators that represent the state to an observer after the entire QLCA process is complete. We now implement our theory to describe a real picture of the brain. In this paper, we limit our discussion to a single neuron, where all brain activities are described as neuron connections.

Dendrites on individual neurons process information by generating electrical input signals. Our proposed model includes the conventional threshold model (Koch et al., 1995); however, because we let the entanglement coefficients be arbitrary, we allow more possibilities.

We consider a network consisting of *N*_*n*_ relevant (i.e., that participate in the process) neurons, where each relevant neuron is denoted by an integer *n* (*n* = 1, 2, …, *N*_*n*_). A dendrite attached to an *n*- relevant neuron is associated with the index δ (δ = 1, 2, …*N*_*d*_). Each δ-dendrite carries a voltage *V*_δ_, which is an additive quantity. Therefore, we can identify a δ-dendrite that carries an impulse *V*_δ_ with a state |*V*_δ_〉. Thus, we can define state products as

(21) $$\begin{array}{l} |V\rangle_{n}=\underset{\delta }{\prod }|V_{\delta }\rangle_{n}, \end{array}$$

where *V* denotes the total voltage generated among all dendrites. The neurotransmitters that arrive at a dendrite interact with receptors and spread along the dendrite surface. We recall that the output voltage of a receptor is not fixed. It depends on the type of neurotransmitter, such that a receptor that interacts with a neurotransmitter to generate a voltage firing tendency can inhibit firing when interacting with another neurotransmitter. In other words, different types of activities that evolve various types of neurotransmitters can generate different values of *V*_*n*_. Consequently, an *n*-neuron possesses two contrasting groups of |*V*〉_*n*_, exciting or inhibiting states. By associating each group with QLCA rules and following our previous discussion, we can identify each group with an entangled state as follows:

**Excitation states that allow the neuron to fire (ϵ and** ***E*** **for excitatory)**

(22) $$\begin{array}{l} |\epsilon \rangle_{n}=\underset{V}{\sum }E_{V}|V\rangle_{n}, \end{array}$$

**Inhibitory states that prevent the neuron from firing (ι and** ***I*** **for preventing)**

(23) $$\begin{array}{l} |\iota \rangle_{n}=\underset{V}{\sum }I_{V}|V\rangle_{n}, \end{array}$$

where *E* and *I* are entanglement coefficients. Note that because the overlap between these groups is forbidden (a neuron cannot excite or inhibit an impulse simultaneously), |ϵ〉_*n*_ and |ι〉_*n*_ are orthogonal.

By denoting the misfire and fire activities of an *n*-neuron as |0〉_*n*_ and |1〉_*n*_, we can express the QLCA states as

(24) $$\begin{array}{c} \text{States for set A}\\ Excitation\;states\;|a_{I}\rangle_{n}=|0\rangle_{n}|\epsilon \rangle_{n}\\ Inhibitory\;states\;|a_{II}\rangle_{n}=|1\rangle_{n}|\iota \rangle_{n} \end{array}$$

(25) $$\begin{array}{c} \text{States for set B}\\ Excitation\;states\;|b_{I}\rangle_{n}=|1\rangle_{n}|\epsilon \rangle_{n}\\ Inhibitory\;states\;|b_{II}\rangle_{n}=|0\rangle_{n}|\iota \rangle_{n}, \end{array}$$

to yield the QLCA operators for an *n*-neuron.

- **Set A**
  – **QLCA-observable of an** ***n*****-neuron**,
    (26) $$\begin{array}{l} {𝕆}_{n}^{\left\{A\right\}}=|a_{I}\rangle_{n}\langle a_{I}{|}_{n}+\alpha_{II}|a_{II}\rangle_{n}\langle a_{II}{|}_{n} \end{array}$$
  – **Correction operator for set A**
    (27) $$\begin{array}{l} {\mathbb{C}}_{n}^{\left\{A\right\}}=|a_{I}\rangle_{n}\langle b_{I}{|}_{n}+|a_{II}\rangle_{n}\langle b_{II}{|}_{n} \end{array}$$
- **Set B**
  – **QLCA-observable of an** ***n*****-neuron**,
    (28) $$\begin{array}{l} {𝕆}_{n}^{\left\{B\right\}}=|b_{I}\rangle_{n}\langle b_{I}{|}_{n}+\beta_{II}|b_{II}\rangle_{n}\langle b_{II}{|}_{n} \end{array}$$
  – **Correction operator for set B**
    (29) $$\begin{array}{l} {\mathbb{C}}_{n}^{\left\{B\right\}}=|b_{I}\rangle_{n}\langle a_{I}{|}_{n}+|b_{II}\rangle_{n}\langle a_{II}{|}_{n} \end{array}$$

If we could implement the CA evolution to describe neuron behavior, we would probably find CA systems that reach a steady phase. In the simplest scenario, they would probably belong to class 1 type (see for example, Wolfram, 1983). This study focuses only on a single neuron activity, while excluding interactions between different neurons. Hence, tools for tracking this CA evolution are lacking. Nevertheless, we can still implement the operator's tools for the toy model proposed in section 3.2 if we consider that after sufficiently large *k* iterations (see Equation 20), the system reaches a steady phase. For this scenario, we can describe the brain activity in operator form as

(30) $$\begin{array}{l} {𝔸}_{n}={𝕆}^{\left\{A\right\}}{\left({\mathbb{C}}^{\left\{A\right\}}\right)}^{k}\\ {𝔹}_{n}={𝕆}^{\left\{B\right\}}{\left({\mathbb{C}}^{\left\{B\right\}}\right)}^{k} \end{array}$$

with

(31) $$\begin{array}{l} {𝕆}^{\left\{A\right\}}=“\sigma_{A}“\underset{n}{\sum }{𝕆}_{n}^{\left\{A\right\}}\;{𝕆}^{\left\{B\right\}}=“\sigma_{B}“\underset{n}{\sum }{𝕆}_{n}^{\left\{B\right\}}\\ {\mathbb{C}}^{\left\{A\right\}}=\underset{n}{\sum }{\mathbb{C}}_{n}^{\left\{A\right\}}\;{\mathbb{C}}^{\left\{B\right\}}=\underset{n}{\sum }{\mathbb{C}}_{n}^{\left\{B\right\}} \end{array}$$

where the eigenconcepts σ describe the manner in which the observer experiences the process. Note that in all cases, the *n* summations were over the relevant neurons.

### 4.1. Threshold Model

An acceptable model for a neuron to be excited is that the sum of the voltages provided by the dendrites will pass a threshold *V*_*m*_ (Koch et al., 1995). This does not mean that to excite a neuron, all dendrites should have their maximum potential. In fact, there are many possibilities for reaching this threshold potential. Because each combination is described by a product of the dendrite states, and there are many combinations of dendrite voltages that can exceed *V*_*m*_, all products will be in a superposition to form an entangled state to define a QLCA rule. If all combinations contribute equally to firing the neuron, the coefficients *E*_Δ_ or *I*_Δ_ in Equations (22)–(23) will be identical. Different coefficients indicate that dendrites with high coefficients have priorities for influencing neuron activity.

## 5. Discussion

In this paper, we showed that the decision for a neuron to fire or misfire is controlled and managed by rules that can be represented mathematically by the QLCA processes. Implementing a mathematical model in brain research corresponds to the definition of parameters, which in our description are the entanglement coefficients. These parameters can characterize various processes and have the potential to denote the associated brain diseases as anomalies in the magnitude of the coefficient. In the threshold model, where all coefficients are equal, even a few unequal parameters can indicate abnormal functioning. Our formalism, which aimed to describe a single neuron reaction, can be extended to describe further brain processes, such as the behavior of dendritic spines (Popovic et al., 2015; Beaulieu-Laroche et al., 2019; Roome and Kuhn, 2019): dendritic spines are small protrusions on the surface of dendrites (Irie and Yamaguchi, 2009) that generate a pulse by interacting with a neurotransmitter. By assuming that the total magnitude of a dendritic pulse is determined by rules imposed on the individual's neurotransmitter-receptor outputs, it is possible to impose a QLCA formalism to describe the output of a dendritic spine. Note that the current understanding of the electrical behavior of dendritic spines is limited because it is difficult to record input signals with satisfactory resolution (Popovic et al., 2015; Beaulieu-Laroche et al., 2019; Roome and Kuhn, 2019). Until proven wrong, we can consider the complicated behavior of rules that may include entanglement with unequal coefficients.

We showed that for each QLCA activity, there are two “mirror” sets, referred to as set A and set B. From a mathematical and logical point of view, the existence of two alternatives (fire or misfire) enforces the existence of two sets. However, if we observe the threshold model, it seems more reasonable that a neuron will fire when the total volume reaches a threshold voltage, rather than stopping firing. Although mathematically, we obtain two alternatives for each action, and reality probably forces us to consider only one side of the mirror.

## Data Availability Statement

The original contributions presented in the study are included in the article/supplementary material, further inquiries can be directed to the corresponding author/s.

## Author Contributions

The author confirms being the sole contributor of this work and has approved it for publication.

## Conflict of Interest

The author declares that the research was conducted in the absence of any commercial or financial relationships that could be construed as a potential conflict of interest.
